# Exfoliated MoS_2_ Sheets and Reduced Graphene Oxide-An Excellent and Fast Anode for Sodium-ion Battery

**DOI:** 10.1038/srep12571

**Published:** 2015-07-28

**Authors:** Tuhin Subhra Sahu, Sagar Mitra

**Affiliations:** 1 Electrochemical Energy Laboratory, Department of Energy Science and Engineering Indian Institute of Technology Bombay, Powai, Mumbai 4000 76, Maharashtra, India

## Abstract

Three dimensional (3D) MoS_2_ nanoflowers are successfully synthesized by hydrothermal method. Further, a composite of as prepared MoS_2_ nanoflowers and rGO is constructed by simple ultrasonic exfoliation technique. The crystallography and morphological studies have been carried out by XRD, FE-SEM, TEM, HR-TEM and EDS etc. Here, XRD study revealed, a composite of exfoliated MoS_2_ with expanded spacing of (002) crystal plane and rGO can be prepared by simple 40 minute of ultrasonic treatment. While, FE-SEM and TEM studies depict, individual MoS_2_ nanoflowers with an average diameter of 200 nm are uniformly distributed throughout the rGO surface. When tested as sodium-ion batteries anode material by applying two different potential windows, the composite demonstrates a high reversible specific capacity of 575 mAhg^−1^ at 100 mAg^−1^ in between 0.01 V–2.6 V and 218 mAhg^−1^ at 50 mAg^−1^ when discharged in a potential range of 0.4 V–2.6 V. As per our concern, the results are one of the best obtained as compared to the earlier published one on MoS_2_ based SIB anode material and more importantly this material shows such an excellent reversible Na-storage capacity and good cycling stability without addition of any expensive additive stabilizer, like fluoroethylene carbonate (FEC), in comparison to those in current literature.

**C**ompare to lithium-ion battery, sodium-ion battery is more compatible to being part of a large-scale storage system that stores energy for renewable energy such as solar and wind where energy is produced intermittently. Although Li-ion batteries are high energy density batteries that can store large amount of energy in a small size however, the negative aspects associated with this technology like high cost, cycle life and safety restrict them to apply universally. On the other hand, sodium is the sixth most common element on the planet and easy to extract from earth crust, therefore batteries can be made of sodium-ion will be much inexpensive compare to lithium^1^,^2^,^3^,^4^.

However, few challenges that sodium-ion battery facing currently are selection of intercalation/conversion/alloy based anode, electrolyte and electrode-electrolyte interface stability. These problems may arise from an inherent characteristic of sodium, that sodium- ions (1.02 Å) are nearly twice as large as lithium-ions (0.59 Å) and the large size causes a greater change in the host structure upon insertion and de-insertion, which results in a massive failure in cyclic stability in all tested anode materials till date^5^. To date, several attempts have been devoted to develop the electrode materials with improved sodium (Na)-ion intercalation and transportation behaviour. For anode, different kind of materials have been investigated such as, i) non-graphitic carbon anode including hard carbon^6^, carbon microspheres by pyrolysis of polymeric resin^7^, N-doped porous carbon fibres^8^ ii) Ti-based anode materials like, amorphous TiO_2_^9^, Na_2_Ti_3_O_7_/Carbon black^10^ and expanded graphite^11^. Here, all of these anodes involved in a reversible sodium intercalation/de-intercalation mechanism, demonstrating specific capacity in the range of 100–300 mAhg^−1^. Organic electrodes such as, polytriphenylamine^12^, di-sodium terephthalate (Na_2_C_8_H_4_O_4_)^13^ were also reported. Apart from these, alloying/de-alloying type anodes like, SnSb/C^14^ and P/C^15^,^16^ etc. were shown as promising candidates due to their high specific capacity. However, these materials suffer from severer volume expansion (up to 500%) during sodium uptake, causing pulverization of materials thus irreversible capacity loss. Moreover, some of the metals are toxic and some of them leave flammable side products during charge-discharge reaction with electrolyte. On the other hand, metal oxides/chalcogenides have established themselves as potential anodes for SIBs due to their rich electrochemistry and significant high capacity value^17^,^18^,^19^,^20^,^21^,^22^,^23^,^24^.

Molybdenum sulphide (MoS_2_) possesses a typical graphite-like layered structure, where each Mo atom is covalently bonded to S atoms forming two-dimensional S-Mo-S sandwich like structure. Furthermore, these 2D layers are stack together by weak van der Waals attraction providing a large interlayer spacing (0.615 nm vs. 0.335 nm of graphite) along C-axis which can eventually accommodate large Na-ions. However, due to their large surface energy, these 2D nanomaterials have a tendency to restack in order to minimize the surface energy^25^,^26^. Moreover, these materials exhibit low inherent electronic conductivity which also affects their electrochemical performance of Na-ion storage. However, owing to such difficulties, these layered materials can be composite with reduced graphene oxide (rGO). The rGO sheets not only improve the electrical conductivity, and the same enhances the mechanical strength. Furthermore, rGO sheets can act as a spacer that can inhibit further agglomeration of MoS_2_ nano-sheets.

Most of the previous reports, MoS_2_ nanoflowers were prepared by hydrothermal synthesis route, result in formation of MoS_2_ with particle size ranges between 500 nm to 3 μm with lattice fringes thickness ≥12 nm^27^,^28^,^29^,^30^.Therefore it is obvious that for sodium battery application, those bigger particle will significantly increase the Na-ion diffusion path and thick MoS_2_ plates will suffer from more internal strain during reaction with sodium, leading to cracking of material, hence poor electrochemical performance. Therefore, it is better to develop a strategic synthetic route that will produce ultrathin MoS_2_ layers with smaller particle size.

Herein, we report a simple hydrothermal technique to produce MoS_2_ nanoflowers with optimized stabilizer (PVP) concentration and low hydrothermal temperature. The low hydrothermal temperature promotes the slow nucleation of MoS_2_ nanoparticles to form controlled MoS_2_ nanosheets and PVP assists the controlled growth of those nanosheets. Further, a composite of exfoliated MoS_2_ nanoflowers with rGO has been constructed by simple liquid phase exfoliation method. The exfoliated MoS_2_ nanoflowers and graphene composite could exhibit excellent electrochemical performance against sodium compare to most of the recent literature.

## Results and Discussion

**Figure 1:**
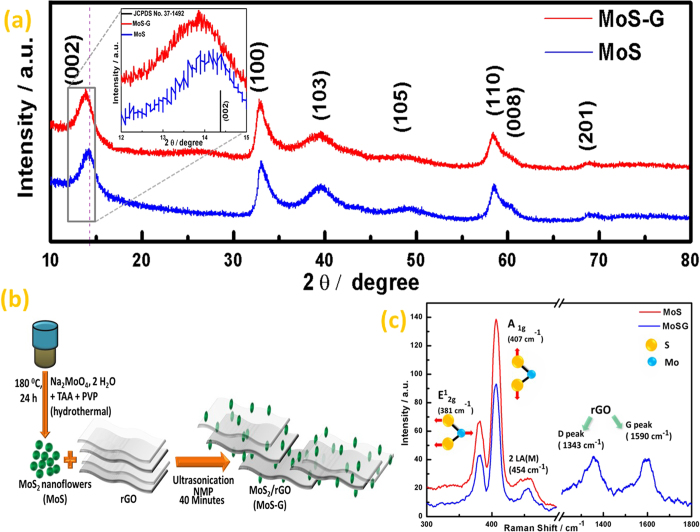
Structural characterization of MoS and MoS-G samples. (**a**) X-ray diffraction (XRD) pattern of MoS and MoS-G, (**b**) Schematic representation of MoS_2_ nanoflowers (MoS) and MoS_2_/rGO composite (MoS-G) preparation, (**c**) Raman spectra of MoS and MoS-G.

Figure 1a, represents the X-ray diffraction (XRD) pattern of as synthesized MoS_2_ nanoflowers (referred as MoS hereafter) and MoS_2_/rGO (referred as MoS-G hereafter) demonstrate phase purity of MoS_2_ with hexagonal structure (JCPDS # 37-1492). In both cases, peak broadening imply the less number of stacked, disordered layers with smaller crystallite size. Moreover, a shift in (002) diffraction peak from 2θ = 14.04^0^ in MoS to 2θ = 13.8^0^ in MoS-G demonstrate there is an increase in interlayer spacing of (002) crystal plane for MoS-G as compared to MoS. Magnified view of (002) peak is shown in inset of Fig. 1a with (002, 2θ = 14.378) of JCPDS # 37-1492 as a standard. Furthermore, a broadened peak appearing at 2θ = 26.14^0^ in case of MoS-G, is purely associated to (002) plane of rGO, which verifies the presence of rGO in MoS-G sample. However, no shift in 2θ was observed for the remaining peaks of MoS and MoS-G. Further, to know the purity of MoS and MoS-G, Raman spectroscopy was carried out. The Raman Spectra of MoS and MoS-G were shown in Fig. 1c. The three peaks in the lower wave number range (300–500 cm^−1^) correspond to hexagonal MoS_2_. The peak at 381 cm^−1^ ascribed to E^1^_2g_, associated with the opposite vibration of two S atoms with respect to Mo atom and A_1g_ peak at 407 cm^−1^, are originated from out-plane vibration of only S atoms^31^. Another typical MoS_2_ peak, assigned as 2 LA (M) appears at 454 cm^−1^. Whereas, MoS-G exhibits two additional broad peaks around 1343 cm^−1^ and 1590 cm^−1^, attributed to D-band and G-band of rGO arise from defect-induced vibrations and in-plane vibrations of Sp^2^ hybridized carbon in rGO.

**Figure 2:**
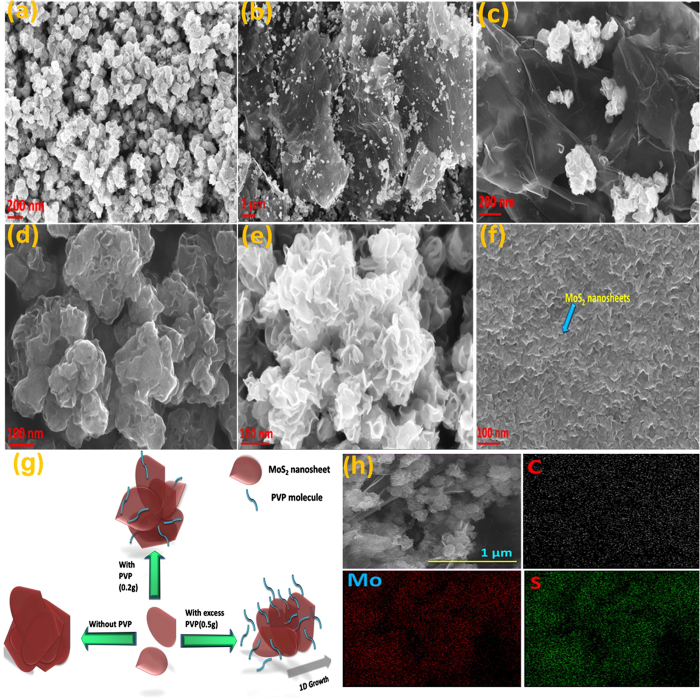
FEG-SEM images of MoS and MoS-G samples. (**a**) MoS, (**b**,**c**) MoS-G at different magnifi-cations, (**d**) MoS (without PVP), (**e**,**f**) MoS synthesized with optimum and excess PVP ratio respectively. (**g**) Schematic illustration of effect of PVP concentration on morphology of MoS_2_ nanosheets. (**h**) Elemental mapping of MoS-G.

**Figure 3:**
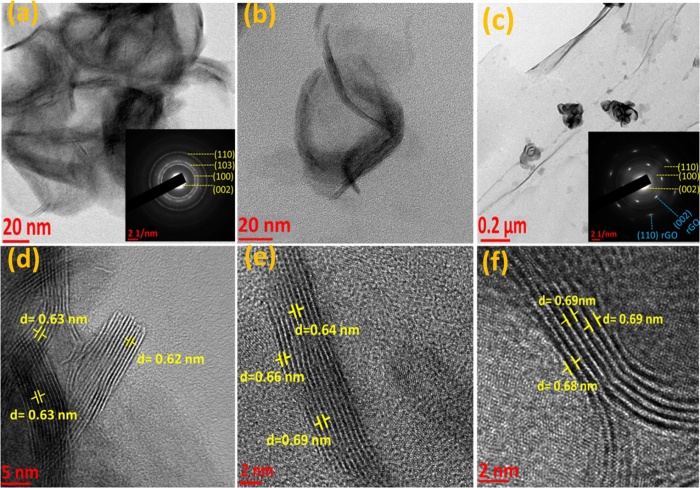
TEM analysis of MoS and MoS-G. (**a**) MoS (inset: SAED pattern), (**b**) MoS after 30 minutes sonication, (**c**) MoS-G along with SAED pattern as inset. (**d**–**f**) HRTEM images of MoS before, after 30 minutes and 40 minutes of sonication respectively.

The morphology and microstructural analysis of as prepared MoS_2_ and its composite with rGO were carried out by field emission scanning electron microscopy (FE-SEM), transmission electron microscopy (TEM). Figure 2a shows flower-like three-dimensional nanosphere architecture of MoS_2_, with a diameter ranging between 100–300 nm. The close-up view of MoS_2_ nanoflowers shown in Fig. 1e depicts these nanoflowers consist of ultrathin nanosheets with a thickness of less than 8 nm. Furthermore, in order to investigate the role of PVP during hydrothermal synthesis, MoS_2_ has been synthesized with excess amount (0.5 g of PVP) of PVP and without PVP under the same experimental conditions. It is observed that without PVP, the irregular MoS_2_ nanospheres were formed, where individual nanosheets are rarely observed (Fig. 2d). While, by using excess PVP there was no sphere formation instead one dimensional growth of MoS_2_ nanosheets was observed (Fig. 2f). Therefore it can be concluded that, PVP plays an important role in forming such smaller size and regular shape of MoS_2_ nanoflowers. From morphological study, it can be said that PVP could be adsorbed on MoS_2_ surface through its C-N and C=O interaction when sufficient amount was used, which prevented the agglomeration of MoS_2_ nanosheets further and facilitated a controlled three dimensional growth of nanosheets. Without any capping agent like PVP, irregular growth of MoS_2_ nanosheets was observed and resulted in agglomerated nanostructures. Further, with increasing the PVP concentration, the three dimensional growth of MoS_2_ nanosheets was supressed by the presence of excess amount of PVP in the solution; hence one dimensional growth was predominantly observed (Fig. 2f). A schematic presentation of such phenomenon is given in Fig. 2g. As indicated by Fig. 2b,c and S1, individual MoS_2_ nanoflowers with an average diameter of ≤200 nm are uniformly distributed on rGO surface. For microstructural analysis, transmission electron microscopy (TEM) experiments were performed and the images provide more detailed structural information of MoS_2_ nanoflowers and MoS_2_/rGO composite. Figure 3a, represents the TEM image of as-prepared MoS_2_ nanoflowers, and inset shown a distinct ring pattern of selected-area electron diffraction (SAED) further demonstrates that it is well-crystallized. Figure 3b, displays micrographs of MoS_2_ nanoflowers after 30 minute of sonication in NMP solvent. From these two figures, it is visible that after sonication, the agglomeration size of MoS_2_ nanoflowers significantly decreased compare to as-prepared MoS_2_ nanoflowers and the transparency level also increased. This reflects relatively loose packing with decreased number of MoS_2_ nanosheets after ultrasonic treatment. Figure 3c depicts TEM image of MoS_2_/rGO composite after 40 minutes of sonication along with rGO, where individual MoS_2_ nanoflowers with expanded interlayer spacing are uniformly distributed on rGO matrix. The SAED pattern of MoS_2_/rGO composite shown as inset of Fig. 3c. Furthermore, high resolution transmission electron microscopy (HR-TEM) images reveal the effect of sonication time on the interlayer distance of MoS_2_ nanoflowers in NMP. It has been found that MoS_2_ nanoflowers, initially having an interlayer distance of 0.62–0.63 nm (Fig. 3d) increased to 0.64–0.69 nm (Fig. 3e) only after 30 minutes of sonication. Moreover, ultrasonic treatment for additional 10 min results in an interlayer spacing of 0.68–0.69 nm (Fig. 3f). At the same time, it is also observed that the number of layers in MoS_2_ nanoflowers gradually decreased with increasing the sonication time.This may be due to the chipping off of 2D nanosheets from the outer surface of MoS_2_ by vibration induced by ultrasonication^32^,^33^.

The electrochemical performance of MoS_2_ nanoflowers and MoS_2_/rGO (MoS-G) electrodes was evaluated using Swagelok type half-cell configuration with Sodium metal as counter/reference electrode. Figure S2a and S2b (as supporting information) display the cyclic voltammograms (CV) for the first five cycles of MoS_2_ nanoflowers and MoS-G electrodes at a scan rate of 0.1 mV.S^−1^ with a potential window ranging between 0.001–2.6 V. In the first cathodic sweep, MoS_2_ nanoflower shows three reduction peaks at around 0.79, 0.61 and 0.005 V. Their corresponding oxidation peaks appeared at around 2.24, 1.80 and 0.45 V. From second cycle, the reduction peaks at 0.61 and 0.79 V shift to higher potential of 0.7 and 1.4 V, respectively. Second cycle onwards, the stability of CV peaks in the subsequent cycles demonstrates a highly reversible and stable sodiation and desodiation process. The reduction peak at 1.4 V can be ascribed as intercalation of sodium-ions into MoS_2_ layer^34^ while the peak at 0.7 V can be ascribed to the conversion reaction.

Therefore as per the study, the overall reaction can be represented as^35^:




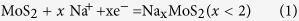







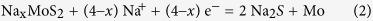




**Figure 4:**
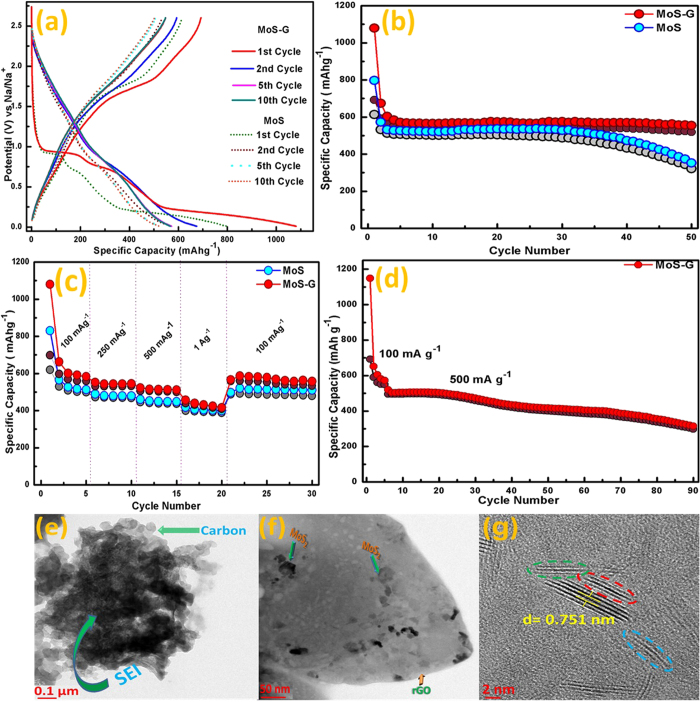
Electrochemical performances of MoS and MoS-G via conversion reaction. (**a**) Charge-discharge profile at 100 mAg^−1^ between 2.6 V–0.01 V vs. Na/Na^+^, (**b**) Cycling performance electrodes at 100 mAg^−1^, (**c**) Power cycle at different current rates, (**d**) Cycling performance of MoS-G anode at 500 mAg^−1^. (**e**,**f**) Low-magnification TEM images of MoS and MoS-G electrode after 50^th^ charge cycle respectively, and (**g**) HR-TEM image of MoS-G after 50^th^ charge cycle.

The galvanostatic charge-discharge performance of MoS and MoS-G electrodes was further evaluated using Swagelok type half-cell configuration. Figure 4a, represents the voltage vs. capacity curves when both the electrodes were charged-discharged at 100 mAg^−1^ current rate within a potential window of 2.6 V–0.01 V vs. Na/Na^+^. In both the cases, 1^st^ discharge curve shows three plateaus at 1.0–0.8 V, 0.75 V–0.4 V and 0.4–0.01 V which are consistent with the CV analysis. Figure 4b demonstrates the cyclic stability of MoS and MoS-G at 100 mAg^−1^ in the same potential range. MoS electrode displays a discharge capacity of 797 mAhg^−1^ in 1^st^ cycle and an average capacity of 528 mAh g^−1^ was observed after 30 cycles. But, after 30 cycles a gradual reduction in discharge capacity (i.e.528 mAh g^−1^ to 373 mAh g^−1^ at the end of 50 cycles) as well as in the coulombic efficiency were observed. To improve the cyclic stability of MoS_2_ nanoflowers, a composite material was prepared with rGO (5% of total MoS_2_ content). MoS_2_ nanosheets/graphene composite (MoS-G) delivered initial discharge capacity of 1080 mAh g^−1^ and a reversible capacity of 575 mAh g^−1^ was observed after 10 cycles. At the end of 50 cycles, a discharge capacity of 557 mAh g^−1^ was retained, which is about 97% of the reversible capacity of 3^rd^ cycle. A coulombic efficiency of around 96% was estimated for initial 30 cycles, which decreased to 94% after 50 cycles. Figure 4c, displays the rate performance of MoS and MoS-G electrodes at different current densities where both the electrodes showed excellent reversible capacity at high current densities (i.e. 500 mA g^−1^ and 1 A g^−1^). At 1 A g^−1^, their respective discharge capacities were 406 mAh g^−1^ and 433 mAh g^−1^, which is about 75–78% of their discharge capacity at 100 mA g^−1^. Furthermore, when the current density reversed to 100 mA g^−1^, both the electrodes recovered their initial capacity after 20 cycles, suggesting an excellent rate performance cycling stability and robustness of the electrode. The rate capability of MoS electrode can be attributed to its small particle size (≤300 nm) and ultrathin lattice fringes (≤8 nm) that significantly reduces the Na-ion diffusion path. Recently, Wang *et al.* have shown rGO can deliver a reversible capacity of ~176 mAh g^−1^ at 100 mA g^−1^ against sodium^36^. Therefore 5 wt% rGO in the composite (MoS-G) is expected to contribute only ~11 mAh g^−1^. Hence, it can be concluded that the rGO present in the composite is not contributing significantly to the capacity; rather it improves the interfacial electron transfer in between MoS_2_ and rGO surfaces during charge/discharge process. Further rGO induces an increased porosity and high surface area of the composite material^37^,^38^ which can facilitate transport of solvated Na-ion through the electrode, as a result of which, Na^+^ can reach each part of electrode materials. rGO can also act as a mechanical buffer to ensure that no change in the electronic connectivity of MoS_2_ nanoflowers occurs due to the volume change encountered during sodiation/desodiation and helps to maintain the structural integrity of the composite electrode. Additionally, in case of any fracturing due to cycling fatigue, incorporation of rGO in the composite ensures the fractured particle remains in electronic circuit and ensures better capacity retention. Further, to understand the reason behind gradual capacity fading of MoS electrode after 30 cycles and how very less amount of rGO is improving the electrochemical performance of MoS_2_ in MoS-G composite, ex-situ TEM analysis of electrodes was performed after their corresponding 50^th^ charge cycle. As indicated in Fig. 4e, no trace of MoS_2_ nanoflowers can be found after 50^th^ charge cycle. It might be due to pulverization of electrode material that results in the formation of very fine MoS_2_ nanoparticles which further masked by additive carbon and SEI layer formed during charge/discharge cycling. Such phenomenon leads to loss of contact with current collector hence rapid capacity fading. However, in presence of rGO, even after 50 cycles MoS_2_ nanoplates with lateral length 5–20 nm were observed on rGO surface (Fig. 4f). HRTEM images of those nanoplates reveal (Fig. 4g), these are mainly single-layer (blue dotted circle), bi-layer (green dotted circle) or tri-layer (red dotted circle) MoS_2_ nanosheets with expanded interlayer spacing. Thus, rGo possibly helps in keeping MoS_2_ nanosheets a part of the electrical circuit even after fracturing/pulverisation.

The long-term stability of the electrochemical performance of MoS-G was further evaluated at a high current rate of 500 mA g^−1^. The MoS-G electrode was first discharged at 100 mA g^−1^ for initial five cycles, and then at 500 mA g^−1^ for 90 cycles. It is evident from Fig. 4d, when discharged at a very high current density i.e. 500 mA g^−1^, MoS-G exhibits a capacity of ~525 mAh g^−1^ for 1^st^ cycle and retains a capacity of 318 mA g^−1^at the end of 90 cycles. The Coulombic efficiency is in the range of 94–98%.

**Figure 5:**
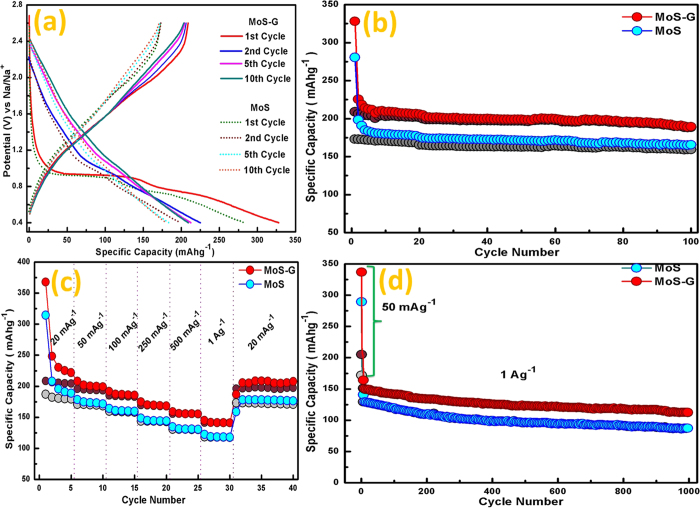
Electrochemical performances of MoS and MoS-G via intercalation mechanism. (**a**) Charge-discharge voltage curves during initial two cycles at 50 mAg^−1^ between 2.6 V–0.4 V vs. Na/Na^+^, (**b**) Cycling performances at 50 mAg^−1^ (**c**) Rate capability at different current densities, (**d**) Cycling performance at 1 Ag^−1^.

The intercalation behaviour of MoS_2_ nanoflowers (MoS) and exfoliated MoS_2_/rGO (MoS-G) was further investigated by restricting the potential window to 0.4 V in the discharge process. Figure 5a demonstrates the potential vs. capacity curves of MoS and MoS-G at 50 mA g^−1^. It is evident that, 1^st^ discharge curve in both cases consists of two plateaus at around 0.97–0.8 V and 0.8–0.4 V, which further shifted to 1.6–1.1 V and 1.06–0.7 V from 2^nd^ discharge onwards. It reflects higher degree of reversible sodium intercalation/de-intercalation process. According to Fig. 5b, MoS-G, when charged-discharged at 50 mA g^−1^, delivers an initial discharge capacity of 328 mAh g^−1^ and then maintained an average capacity of ~203 mAh g^−1^ for 100 cycles with ~100% Coulombic efficiency. As per our knowledge, this is one of the best results obtained among recently published MoS_2_ SIB anodes based on intercalation/de-intercalation reaction of Na-ion. Whereas, at the same current rate of 50 mA g^−1^, MoS electrode displays an average reversible capacity of ~172 mAh g^−1^ for 100 cycles, but exhibits a relatively low efficiency of 96–98%. However, the superiority of MoS-G over MoS is attributed to expanded and exfoliated MoS_2_ interlayers in MoS-G, which actually provides more active Na^+^ storage sites and low energy barrier of Na^+^-ion to intercalation/de-intercalation. The current densities were also varied to investigate the rate capability of both the samples which are shown in Fig. 5c, where minimal drop in capacities with increased current densities indicates higher degree of reversible Na intercalation/de-intercalation owing to their expanded interlayer spacing. The long-term cycling stability of MoS and MoS-G electrodes at a high current density of 1 A g^−1^ is represented in Fig. 5d. It is evident that MoS-G maintains an average discharge capacity of ~131 mAh g^−1^ over 1000 cycles with 100% Coulombic efficiency. MoS electrode delivers an average capacity of ~118 mA g^−1^ for initial 200 cycles which then reduced to ~90 mAh g^−1^ after 1000 cycles. The electrochemical characterization of 8:1:1 ratio electrodes was also carried out and compared with the results of the 6:2:2 ratio electrodes. The reasons for utilizing 6:2:2 electrodes for this study are discussed in the supplementary information Fig. S3. To understand the interfacial charge transfer kinetics of both of these electrodes, EIS experiments were conducted at OCV (open circuit potential). In the impedance spectra (EIS) of MoS and MoS-G electrodes, (shown in Fig. S4, supplementary information), R_o_ represents the ohmic resistance, R_ct_ for charge transfer resistance coupled with double layer capacitance Q_dl_, shown by semicircle and W, Warburg impedance represented by straight line for solid-state diffusion. MoS-G electrode exhibits lower R_ct_ value compared to MoS electrode, indicating less charge transfer resistance at the electrode/electrolyte interface (values are given in the table as inset of Fig. S4). Furthermore, a large slope of the impedance at low frequency region demonstrates better solid-state diffusion of Na^+^-ions into the MoS-G compare to MoS electrode. This is again attributed to high porosity and large surface area induced by rGO matrix in the electrode structure.

**Figure 6:**
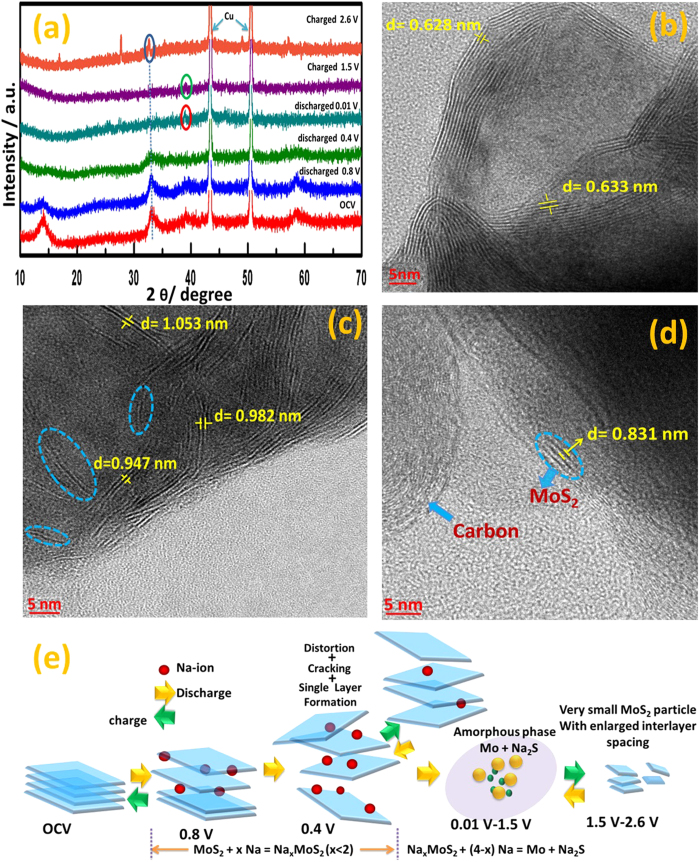
Ex-situ X-Ray diffraction pattern and HRTEM images of MoS electrode. (**a**) Ex-situ X-ray diffraction pattern of MoS at different cut off potentials. (**b**–**d**) HRTEM images revealing structural changes of MoS electrode in 1^st^ cycle at OCV, 0.4 V during discharging, and at 2.6 V during charging respectively, (**e**) Schematic illustration of potential dependence of different stages of reaction.

As we observed in our previous study, MoS_2_ electrode behaves differently with lithium after first discharge-cycle^39^. Using high surface sensitive instruments and with the help of ab-initio calculation we came to a conclusion that in the 1^st^ discharge cycle, MoS_2_ converted to Mo and Li_2_S as usual conversion mechanism. However, during the opposite scan, the reaction changes its route and undergoes alloying reaction between Li and S at room temperature. The extra capacity contribution can be accounted for Li-S alloy reaction and that was not investigated by any other group previously. The proposed mechanism is well agreement with theoretical study and through experimental results. The present study is dedicated to understanding the interaction of Sodium with MoS_2_ in different cycles. In the beginning, the changes in the crystal structure upon sodiation/de-sodiation process were studied using ex-situ XRD taken at various stages of the reaction during first cycle. Figure 6a depicts the XRD pattern of MoS electrode before cycling at open circuit voltage (OCV), at 0.8 V and at 0.4 V in discharge (middle region of the discharge reaction), at 0.01 V after complete discharge, at 1.5 V in the middle of the charge reaction and finally at 2.6 V at the end of the first charge process. When the electrode is discharged to 0.4 V, the diffraction peak corresponding to (002) plane almost disappeared, whereas peaks for (100), (103) and (110) crystal planes became broader. At 0.4 V, MoS_2_ does not involve in any kind of chemical reaction except intercalation of Na^+^ ions into MoS_2_ layers.After complete discharge to 0.01 V, all peaks for MoS_2_ disappeared and only one peak located at 2θ = 38.9^0^(marked in red circle), can be indexed to the (220) plane of Na_2_S (JCPDS No. 03-0933). This phenomenon suggests the amorphous nature of MoS_2_ which converted to Mo and Na_2_S. During charging at 1.5 V, the Na_2_S peak still exists (marked in green circle), but became broader, indicating partial conversion of Na_2_S. When MoS electrode was further charged to 2.6 V, the peak for Na_2_S finally disappeared and a diffraction peak at 2θ = 33^0^ (marked in blue circle) appeared again, which corresponds to (100) crystal plane of MoS_2_, indicating complete conversion of Na_2_S and Mo and reformation of MoS_2_. For more insight of the reaction mechanism, the EDAX and elemental mapping profiles of MoS electrodes at OCV and after completion of 1^st^ cycle (Fig. S5) were performed and revealed the atomic percentage (%) ratio of Mo:S remain constant to 1:2. The observation is also indicative of reformation of MoS_2_ after 1^st^ charge cycle. However, the increased percentage (%) of oxygen along with the introduction of sodium after complete cycle is attributed to surface deposition of Na_2_CO_3_ as SEI and arial oxidation of sodium into Na_2_O during sample handling. To further elucidate the structural changes during charge-discharge process, an ex-situ HRTEM of the MoS electrodes were carried out at three different stages during the 1^st^ cycle: (i) at OCV, (ii) at the end of intercalation reaction i.e. 0.4 V during discharge, (iii) at the end of conversion reaction i.e. 2.6 V after complete charge. It is evident from Fig. 6c, that as the electrode discharged to 0.4 V, the crystallinity of MoS_2_ layers (in Fig. 6b) have been destroyed and a distorted structure with irregular orientation of expanded MoS_2_ nanoplates were observed. Furthermore, such distortion also leads to cracking and formation of single layer MoS_2_ shown by blue dotted circle in Fig. 6c. HRTEM image of charged-discharged electrode at 2.6 V (Fig. 6d) reveals the formation of very fine nanoparticles with enlarged interlayer spacing. The formation of very fine nanoparticles might be the reason for the absence of (002) XRD peak along with other low-intense diffraction peaks of MoS_2_ after a complete charge process.

**Figure 7:**
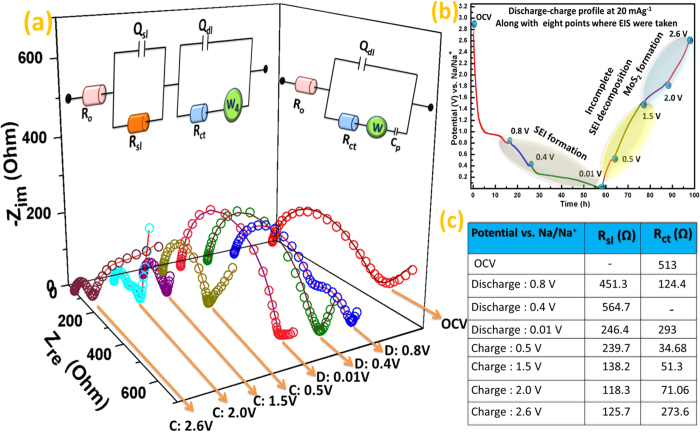
Electrochemical impedance spectroscopy study and analysis of MoS electrode at different state-of-charge conditions. (**a**) EIS (Nyquist plot) spectra of MoS electrode at different potentials during 1^st^ cycle (circles: experimental curve, lines: fitted curve, D: discharge, C: charge, inset: corresponding equivalent circuits), (**b**) charge-discharge profile of 1^st^ cycle along with potential points where EIS were taken, and (**c**) tabulation of impedance values of 1^st^ charge-discharge cycle.

Although several research groups have demonstrated MoS_2_ as potential anode focusing their electrochemical performances, the SEI formation on the electrode surface is still overlooked. Therefore, to further understand the SEI formation, which are mainly Na_2_CO_3_ and alkyl carbonates^40^ on electrode/electrolyte interface during the progress of cell reaction with sodium-ion and the charge-discharge mechanism, in-situ electrochemical impedance spectroscopy (EIS) were performed during 1^st^ discharge-charge cycle and shown in Fig. 7a. The EIS of MoS electrode was taken during sodiation/de-sodiation process at eight specified potentials (Fig. 7b). The analysis of impedance spectra is based on the equivalent circuits presented in the inset of Fig. 7a, where the symbol R_o_, represents the ohmic resistance, contributed by combination of current collectors, electrode, separator and mainly electrolyte, is indicated by the intercept at high frequency. R_sl_ and Q_sl_, represented by semicircle at high frequency are attributed to resistance of migration and capacity of surface-passivation layer respectively. The R_ct_ and Q_ct_ stand for charge transfer resistance and double layer capacitance, respectively, shown by a semicircle in mid-frequency region and Warburg impedance (W), displayed by a straight line at low frequency region, originates from the diffusion of charged species (i.e. solvated Na^+^-ion) through the bulk of the electrode material. Form Fig. 7a, it is observed that upon sodiation from OCV to 0.8 V, two semicircles appeared in the corresponding Nyquist plot, where the high frequency semicircle (HFS) displays high resistive nature compared to medium frequency semicircle (MFS). Generally, the semicircle in high frequency is attributed to the resistance of surface- passivation layer (SEI) and the small semicircle in medium frequency is indexed to the resistance of charge transfer on electrode/electrolyte interfaces^41^. Hence, HFS at 0.8 V can be considered to be originating from SEI formation on the interface (the resistance of SEI film coupled with SEI film capacitance) and the MFS is related to Na- intercalated MoS_2_ on the surface of the electrode. This phenomenon is very similar to Li/graphite system where intercalation-deintercalation is a dominant process^42^. As the electrode discharged to 0.4 V, the small semicircle in the medium frequency region disappeared instead a single semicircle with significant high R_sl_ value was observed. Such phenomenon indicates the formation of a thick SEI layer on the surface. Another reason being that more Na-intercalation at 0.4 V leads to distortion and exfoliation of MoS_2_ (previously shown in ex-situ XRD and ex-situ TEM analysis), providing significant number of fresh electrode/electrolyte interface, on which new SEI layer could be formed. Besides, an almost perpendicular straight line in low frequency region indicates the consumption of Na^+^-ion by SEI layer causing reduction in Coulombic efficiency. This thickening of SEI layer was also verified independently by Lacey *et al.*, via in*-*situ. AFM study where they observed that the average thickness of SEI formed on sodiated MoS_2_ surface is 20.4 nm ± 10.9 nm within a potential window of 3.0 V–0.4 V^43^. Whereas, at 0.01 V, the single semicircle in the whole frequency range is correspondences to the SEI layer dominating impedance response. However, a little decrease in R_sl_ value and slight inclination of Warburg impedance (i.e. solid state diffusion is preferred over Na^+^-ion adsorption) are indicative of formation of a new phase. During charging, upon subsequent de-sodiation, the R_sl_ starts to decrease dramatically and continued to the end of the charge process. Such phenomenon is attributed to dissolution or destruction of SEI layer caused by significant volume change of electrode material due to Na^+^-ion migration^44^,^45^. When the charging process attended to 1.5 V from 0.5 V, there is no significant change in their corresponding Nyquist plots, instead a drop in R_sl_ value corresponding to SEI decomposition was observed. Now, as the electrode charged to 2.0 V, an impedance plot with two semicircles came into scenario. Herein, it is obvious that the semicircle at high frequency is contributed by SEI and the semicircle at medium frequency could be ascribed to incomplete MoS_2_ formation. Moreover, similar phenomenon was also demonstrated by voltage profile charge-discharge curve in Fig. 7b. Where, during charging a voltage plateau starts at 1.5 V and disappeared around 2 V, suggesting main charge reaction happens in this voltage range. Further charging to 2.6 V, the HFS remains almost unaffected, indicating complete destruction of surface-passivating layer is not possible in 1^st^ cycle, where the MFS get depressed and exhibits diffusion dominant behaviour. This is attributed to the formation of very small MoS_2_ particles with expanded interlayer spacing, which significantly increases the surface area of the electrode. Hence, providing sufficient contact between the active material and electrolyte, leads to decrease in charge transfer resistance^41^ which is in good agreement with ex-situ TEM image at 2.6 V (Fig. 6d). Furthermore, formation of such fine nanoparticles induces additional porosity and shorter diffusion pathway allowing Na^+^-ion to diffuse into the bulk.

In summary, a simple and scalable hydrothermal-ultrasonic method has been successfully developed to make exfoliated MoS_2_ and composite of MoS_2_-r graphene oxide as high energy density anodes for SIBs. The as-prepared exfoliated MoS_2_-G electrodes with few-layered of expanded MoS_2_ distributed on the r-graphene films exhibit high capacity, superior rate capability (~570, ~539, ~518 and ~433 mA h g^−1^ at 0.1, 0.25, 0.5 and 1 A g^−1^ respectively. If we restrict the sodium reaction to intercalation then the electrode exhibited ~230, ~203, ~194, ~176, ~165 and ~157 mA h g^−1^ at 0.02, 0.05, 0.1, 0.25, 0.5 and 1 Ag^−1^ respectively), and extraordinary cyclic performance with and within potential cut off. The present study also revel the reaction mechanism of sodium with MoS_2_ and identifies the product and reaction pathways that are quite different from recently reported lithium-ion battery reaction with MoS_2_ by our group. This report also discusses about the SEI formation and its stability and how it’s get affected over cycles as sodium-ion battery anode. Finally, we believe that our study with exfoliated MoS-G anode and fascinating electrochemical properties can alleviate more interesting discussions about the next generation NIB and its potential new applications.

## Methods

### Synthesis of 3D MoS_2_ nanoflowers (MoS)

1.0 g (4.133 mmol) of Na_2_MoO_4_.2 H_2_O was dissolved in 90 mL of DI water followed by addition of 0.2 g of Polyvinylpyrrolidone (PVP). Then, 0.9308 g (12.39 mmol) Thioacetamide (TAA) was added into the same and stirred for 15 min. After that, the whole solution was transferred into a 120 mL Teflon-lined stainless steel autoclave, which was then sealed and maintained at 180 °C for 24 h. After natural cooling of the autoclave, the black coloured precipitates were washed with DI water (**×**2) and absolute ethanol (×1) by centrifugation at 10000 rpm for 5 minutes. Then, the product was dried in an air oven at 60 °C overnight. Finally, MoS_2_ nanoflowers were annealed at 700 °C for 4 h under the flow of 5% H_2_+ 95% N_2_ atmosphere (rate 5 °C/min).

### Synthesis of Graphene Oxide (GO)

GO was synthesized by method as mentioned elsewhere with some modification^46^. Typically, 1 g (1 equiv.) of natural graphite flakes were added into 9:1 mixture of concentrated H_2_SO_4_: H_3_PO_4_ (120:40 mL). After that, 6 g (6 equiv.) of KMnO_4_ was added very slowly about 1 h. Then, the reaction was heated to 55 °C and stirred for 12 h. After formation of a brown colour cake, the whole cake was poured onto ice followed by drop wise addition of 3 mL 20% H_2_O_2_. Now, the colour of the colloidal solution turns into bright yellow. The product then repeatedly washed with copious amount of DI water and absolute ethanol by centrifugation at 5000 rpm for 15 minutes until pH of the supernatant reaches to 7. Finally, GO was dried in an air oven at 80 °C overnight.

### Synthesis of reduced Graphene Oxide (rGO)

rGO has been synthesized by thermal reduction of GO. Simply, 0.1 g GO sheets are thermally treated in a lindberg tube furnace under a stream of Argon gas at 900 °C for 1 h with a heating rate of 5 °C/min.

### Synthesis of 3-D MoS_2_/rGO (MoS-G)

100 mg of as prepared MoS_2_ nanoflowers were added to 100 mL N-methylPyrrolidone (NMP). The mixture then stirred for 15 minutes and kept overnight. After that, the resulting dispersion was sonicated for 30 minutes at room temperature using a probe sonicator (250 W Branson Ultrasonifier, 35% amplitude, Pulse On: 15 sec, Pulse Off: 2 sec).Then, 20 mL of rGO suspension (0.25 mg.mL^−1^) in NMP was added drop wisely into it under vigorous stirring. The whole mixture further sonicated for another 10 minutes where all the parameters remain same. After that, the MoSG composite was separated from NMP by centrifugation at 10000 rpm for 5 minutes and repeatedly washed with DI water and absolute ethanol. Finally, the composite sample was dried in a vacuum oven at 100 °C for 12 h.

### Materials Characterization

The crystal structure and phase of as prepared powder samples were characterized by powder X-ray diffraction (XRD) measurements using Rigaku smartlab X-ray diffractometer equipped with Cu Kα radiation (operation voltage: 40 kV, current: 40 mA, λ = 1.5418 Å). Different vibrational moods of metal-sulphur bonds as well as carbon-carbon bonds were detected by using Raman spectrometer (Horiba Jobin Yvon HR800). Surface morphology of the samples was depicted by field emission scanning electron microscopy (FESEM, Carl Zeiss Ultra 55) and energy dispersive X-ray spectroscopy (EDS, Oxford Instruments). Further, detailed microstructure investigations were carried out using high resolution transmission electron microscopy (HRTEM, JEOL-2100F). The electron diffraction patterns obtained from TEM analysis were indexed with use of SingleCrystal Softwere (CrystalMaker Software Ltd.), whereas, the measurements on TEM image were done using ImageJ tool. For ex-situ XRD and ex-situ TEM analysis of cycled (charge-discharged) electrodes, the cells were first dismantled inside an Ar-filled glove box. The electrodes were then washed with dimethyl carbonate (DMC) to remove NaClO_4_ salt and kept inside the glove box for 12 h. After complete drying, the electrodes were sealed in separate air-tight sample boxes and taken out from glove box for respective XRD analysis. Whereas for TEM analysis, first disperse the electrode materials in isopropyl alcohol inside the glove box, then taken out from glove box and drop casted on Cu-grid followed by 30 minutes sonication.

### Electrochemical cell fabrication and measurements

The MoS electrodes were prepared by mixing of active material with Carbon (Carbon black, C65, Timcal, Switzerland) and carboxymethyl cellulose (CMC) sodium salt in de-ionized water in the wt. ratio 6:2:2. For MoS-G the preferred ratio is 6:1.5:2, in order to keep carbon content fixed to 20%. The electrodes were also fabricated using a ratio of 8:1:1. Then those electrode materials were painted on Cu-foil and dried overnight in an air oven at 65 °C. After complete drying, 10 mm circular discs of electrodes were loaded into Swagelok type half-cell configuration cells for electrochemical testing. The cells were assembled in an Ar-filled glove box (UniLab, Mbraun, H_2_0 ~ 0.5 ppm, O_2_ ~1.0 ppm) with cast discs as working electrode against Na-foil (Alfa Aesar) anode which acts as both counter and reference electrode, with a borosilicate glass fibre (GF/D, Whatman) as separator soaked in 1 M NaClO_4_ in 7:3 (w/w) propylene carbonate (PC): ethylene carbonate (EC) electrolyte. The prepared electrodes were then aged for 6 h before any electrochemical testing to ensure good soaking of electrolyte into electrodes and separator. The typical active material loading of the negative electrodes were in the range of 1.22 ± 0.1 mg.cm^−2^. The Galvanostatic charge-discharge tests were performed with Arbin Instrument, USA (BT2000 model, USA) at various current rates with two different voltage cut off of 2.6 V–0.01 V and 2.6 V–0.4 V (vs. Na/Na^+^).Cyclic Voltammetry (CV) experiments were conducted by measuring *i-V* response at a scan rate of 0.1 mVS^−1^ within potential limit of 2.6 V–0.001 V (vs. Na/Na^+^) using Biologic VMP-3 model, France. The in-situ Electrochemical Impedance Spectroscopy (EIS) was carried out using Biologic VMP-3 model, France. Eight different potential points (OCV, different discharge points as: 0.8 V, 0.4 V and 0.01 V vs. Na/Na^+^ and charge points as: 0.5 V, 1.5 V, 2.0 V and 2.6 V vs. Na/Na^+^) were selected according to voltage profile during charge-discharge curve of MoS electrode for in-situ EIS experiments. At each point potentiostatic EIS was taken within a frequency range of 1 MHz to 10 mHz and with voltage amplitude ∆V = 5 mV. For EIS experiments, charge-discharge experiment of electrodes was carried out at a current density 20 mA g^−1^. All the electrochemical performances were measured at a constant temperature of 20 °C with controlled humidity.

## Additional Information

**How to cite this article**: Sahu, T. S. and Mitra, S. Exfoliated MoS_2_ Sheets and Reduced Graphene Oxide-An Excellent and Fast Anode for Sodium-ion Battery. *Sci. Rep.* **5**, 12571; doi: 10.1038/srep12571 (2015).

## Supplementary Material

Supplementary Information
