# Supplementary material for: Exfoliated MoS2 Sheets and Reduced Graphene Oxide-An Excellent and Fast Anode for Sodium-ion Battery
Source: Sci Rep. 2015 Jul 28;5:12571. doi: 10.1038/srep12571 (PMC4517166; doi:10.1038/srep12571)
Supplement: Supplementary Information [file srep12571-s1.pdf]

Supporting Information:

Exfoliated MoS_2 Sheets and Reduced Graphene Oxide-An Excellent and Fast Anode for Sodium-ion Battery

Tuhin Subhra Sahu and Sagar Mitra*
Electrochemical Energy Laboratory, Department of Energy Science and Engineering
Indian Institute of Technology Bombay, Powai, Mumbai 4000 76, Maharashtra, India

Keywords: Exfoliated MoS_2, Cyclic stability, High energy and power density, Reduced graphene oxide, Sodium battery anode

Email: sagar.mitra@iitb.ac.in
Phone: + 91 22 2576 7849

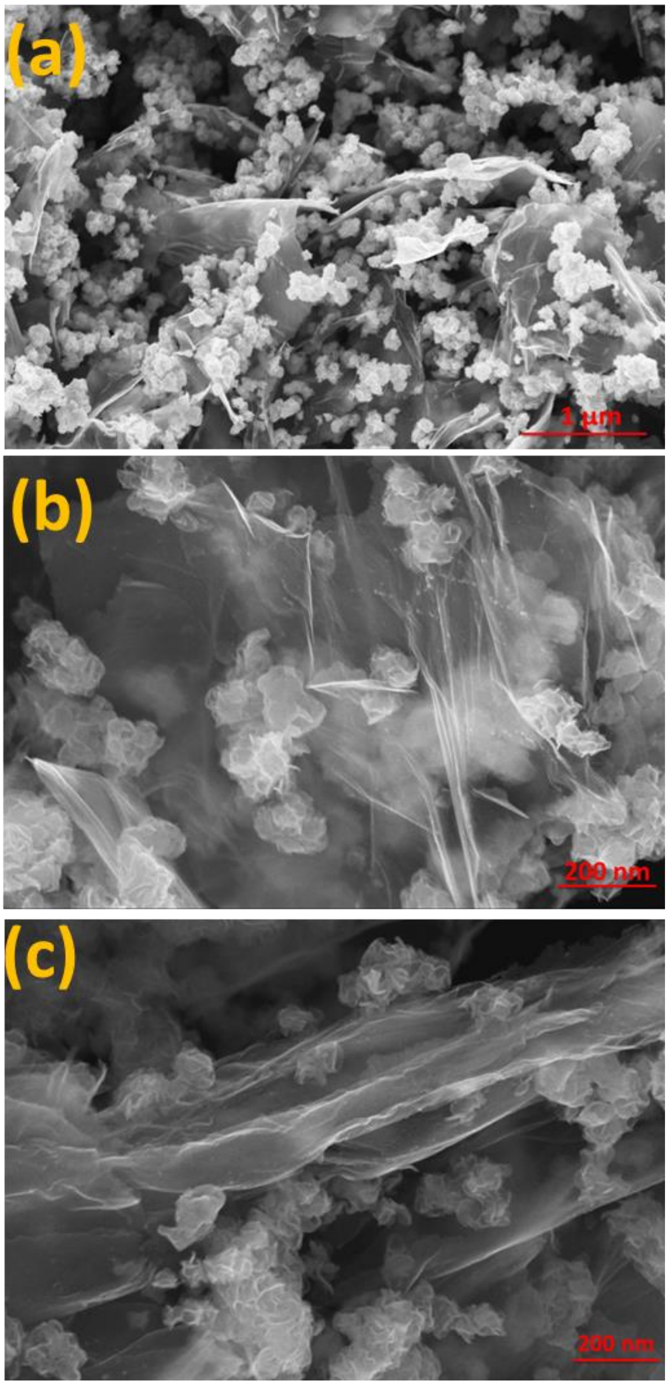

Figure S1. FEG-SEM images of MoS_2-rGO (MoS-G) composite showing the homogenity of the composite and the distribution of nanoflowers on rGO layers at different magnifications.

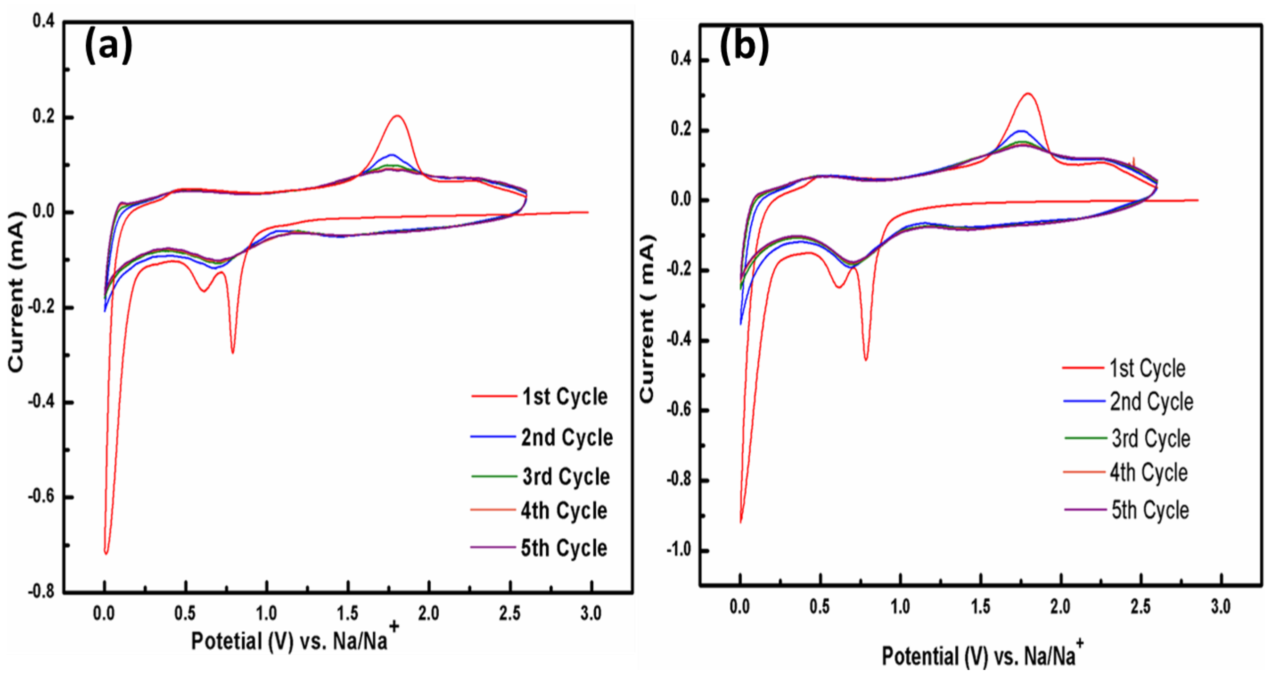

Figure S2. Cyclic Votammograms for the first five cycles of (a) MoS and (b) MoS-G electrodes between 0.001 V-2.6 V with a scan rate of 0.1 mV $s^{-1.}$

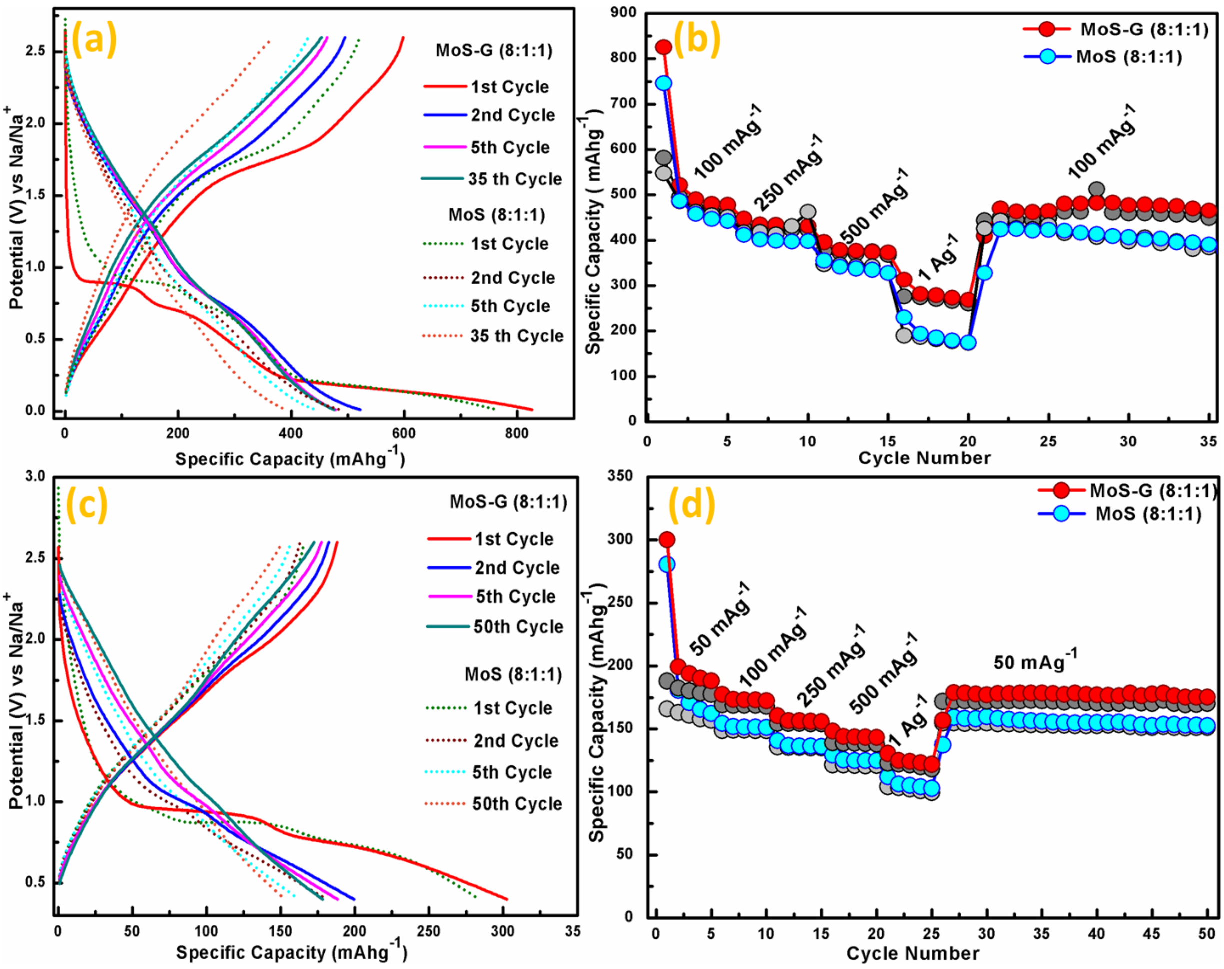

Figure S3. Electrochemical performance of MoS and MoS-G at 8:1:1 ratio. (a-b) and (c-d) galvanostatic charge-discharge profile and rate capibility of MoS and MoS-G electrodes over a potential window of 2.6 V-0.01 vs. Na/Na^+ and 2.6 V-0.4 V vs. Na/Na^+ respectively.

MoS and MoS-G have been tested in 8:1:1 (MoS_2:C:CMC) ratio. From Fig. S4 it is evident that the 8:1:1(MoS_2:rGO+C:CMC) MoS-G electrode delivered a discharge capacity of 486 mAh g^{-1} at 100 mA g^{-1} compared to 575 mAh g^{-1} at same rate when loaded at 6:2:2 (MoS_2:rGO+C:CMC) ratio. Specially at high current density (1 A g^{-1}) 8:1:1 ratio MoS-G displayed 272 mAh g^{-1} which is lower than 433 mAh g^{-1} as observed in 6:2:2 ratio electrode. Thus the performance of the 8:1:1 electrode is

limited by the percolation network of conductive carbon achieved and thus, the results of the 6:2:2 electrodes are a better representation of the material performance. In case of the intercalation behaviour it is observed that the difference between the capacity exhibited by the 8:1:1 and 6:2:2 MoS-G electrodes (~195 mAh g^{-1} vs. 203 mAh g^{-1}) is not as significant as seen in the conversion behaviour. This could be explained via the mechanism of capacity reduction (with reduction of carbon). The capacity reduction observed when less conductive additive is used can be attributed to two reasons: the higher ohmic drop leads to the cut-off conditions being triggered earlier; and the fact that some parts of the electrode may not be perfectly connected by the carbon network due to lack of carbon. In case of MoS-G composite, the second reason is negated due to the extensive and homogenous presence of rGO sheets that ensure proper connection of all MoS_2 nanoflowers. However, the presence of lesser amount of conductive additive ensures a higher ohmic drop and hence, the cut-off conditions will get triggered early even in case of MoS-G composite. However when we compare the drop in capacity in the different voltage windows (intercalation vs. conversion), we observe that the drop in capacity in case of conversion is more than the drop in case of intercalation. This can be explained on basis of the above established mechanism of capacity drop and the nature of voltage profiles towards the end of discharge cycle in the two voltage windows. In case of intercalation, towards the end of discharging, the voltage profile exhibits a sharper slope as compared to the conversion results. Thus, for the same amount of ohmic drop, the material exhibits a larger drop in case of conversion as compared with intercalation.

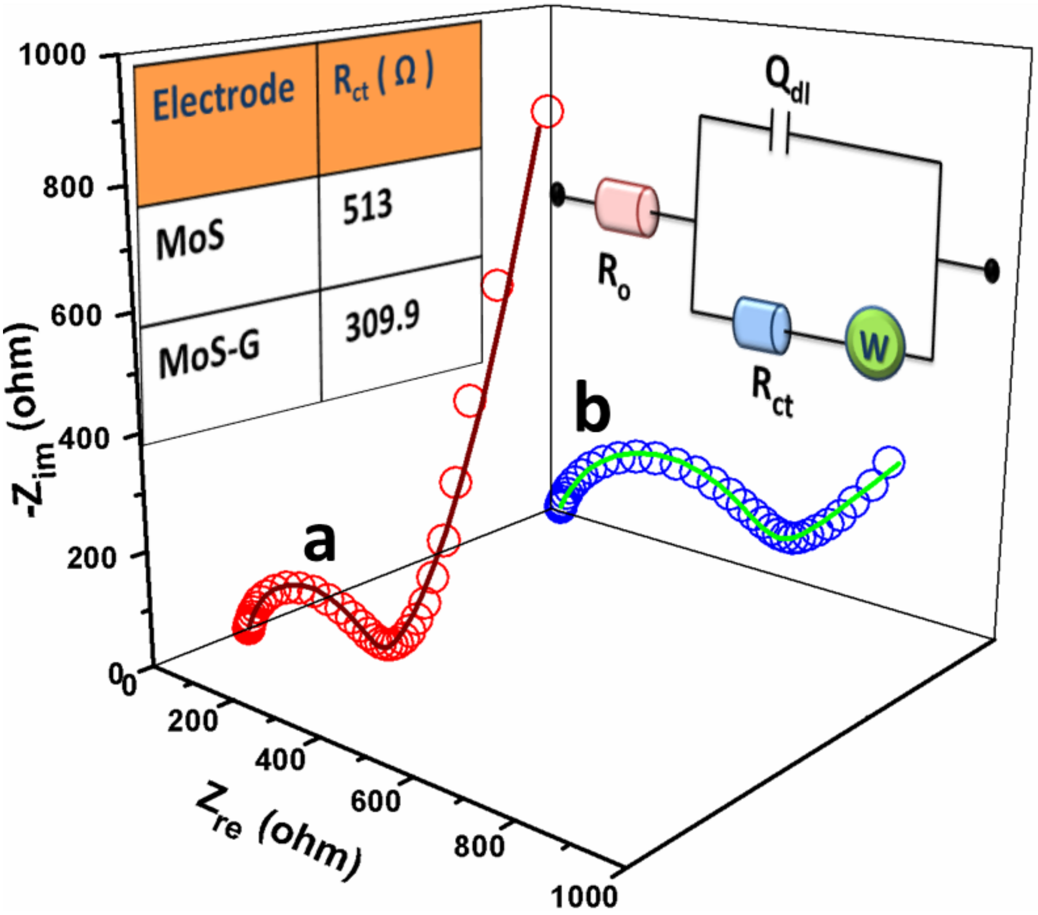

Figure S4. The impedance spectra of (a) MoS-G (b) MoS electrodes in the frequency range between 1 MHz to 10 mHz at 20 ^{0}C (dotted circle: experimental curve, continuous line: fitted curve, Inset: equivalent circuit, tabulated data of R_{ct} value).

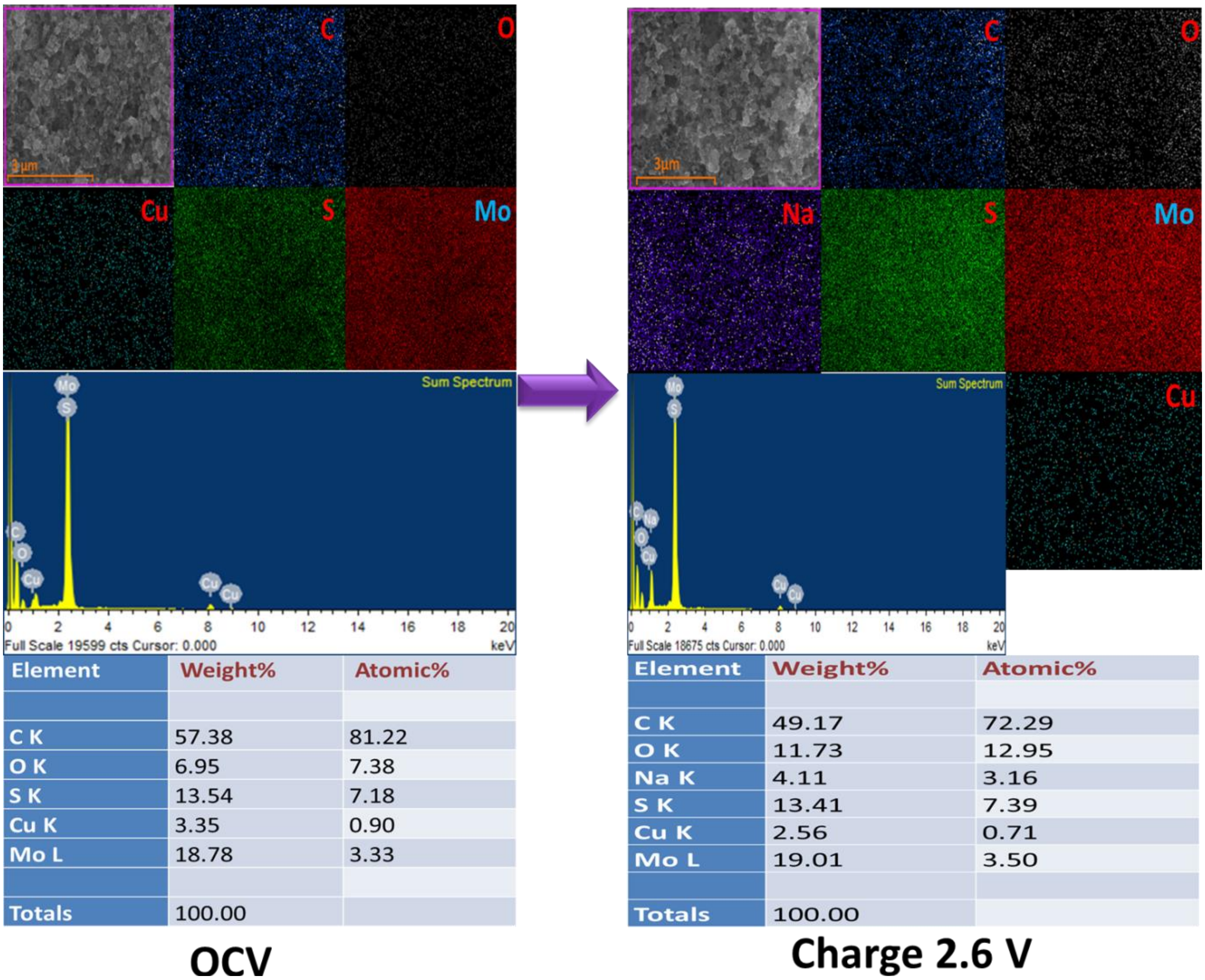

Element	Weight%	Atomic%
C K	57.38	81.22
O K	6.95	7.38
S K	13.54	7.18
Cu K	3.35	0.90
Mo L	18.78	3.33
Totals	100.00	

OCV

Element	Weight%	Atomic%
C K	49.17	72.29
O K	11.73	12.95
Na K	4.11	3.16
S K	13.41	7.39
Cu K	2.56	0.71
Mo L	19.01	3.50
Totals	100.00	

Charge 2.6 V

Figure S5. Elemental mapping and EDAS of MoS electrode showing reformation of MoS_2, (a) OCV, (b) after 1st cycle.

Electrodes	Electrolytes	Specific Capacity ($mAh\ g^{-1}$) Current density ($mA\ g^{-1}$)			References
		Cycle Number			
		1st	10th	50th	
3D MoS_2/rGO (MoS-G)	1 M $NaClO_4$ PC:EC (7:3,w/w)	1080 ($100\ mA\ g^{-1}$) 524 ($500\ mA\ g^{-1}$)	575 ($100\ mA\ g^{-1}$) 511 ($500\ mAg^{-1}$)	557 ($100\ mA\ g^{-1}$) 415 ($500\ mA\ g^{-1}$)	This work
Ultrathin MoS_2 nanosheets	1 M $NaClO_4$ PC:EC (1:1,v/v)	~998 ($40\ mAg^{-1}$)	~520 ($40\ mA\ g^{-1}$)	490 ($40\ mA\ g^{-1}$)	Ref. S1
3D MoS_2 - Graphene microsphere	1 M $NaClO_4$ EC:DMC (1:1,v/v)+ 5 wt% FEC	797 ($500\ mA\ g^{-1}$)	~500 ($500\ mA\ g^{-1}$)	~480 ($500\ mA\ g^{-1}$)	Ref. S2
MoS_2/Graphene	1 M $NaClO_4$ PC:EC (1:1,v/v)	~1700 ($20\ mA\ g^{-1}$)	~650 ($20\ mA\ g^{-1}$)	~580 ($20\ mA\ g^{-1}$)	Ref. S3
MoS_2/C	1 M $NaPF_6$ FEC:DMC (1:1,v/v)	~1500 ($67\ mA\ g^{-1}$)	~540 ($67\ mA\ g^{-1}$)	~520 ($67\ mA\ g^{-1}$)	Ref. S4
3D MoS_2/CNT	1 M $NaPF_6$ EC:DEC:DMC (1:1:1)	835.6 ($50\ mA\ g^{-1}$)	537.3 ($50\ mA\ g^{-1}$)	499.7 ($50\ mA\ g^{-1}$)	Ref. S5
MoS_2 nanosheets (Worm-like structure)	1 M $NaPF_6$ FEC:DMC (1:1, v/v)	675.3 ($61.7\ mA\ g^{-1}$)	~480 ($61.7\ mA\ g^{-1}$)	~430 ($61.7\ mA\ g^{-1}$)	Ref. S6
MoS_2/rGO	1 M $NaClO_4$ PC:EC (1:1,v/v) +5 wt% FEC	~600 ($100\ mA\ g^{-1}$)	~300 ($100\ mA\ g^{-1}$)	~305 ($100\ mA\ g^{-1}$)	Ref. S7

Figure S6. Comparison of our prepared 3D MoS_2/rGO (MoS-G) with previously published MoS_2 SIB anode materials in terms of electrolytes, specific capacity and rate capability.

Electrodes	Specific Capacity (mAh g^{-1})<br>Current density (mA g^{-1})				References
	Cycle Number				
	2nd	10th	50th	100th	
3D MoS_2/rGO (MoS-G)	225<br>(50 mA g^{-1})	210<br>(50 mA g^{-1})	207<br>(50 mA g^{-1})	195<br>(50 mA g^{-1})	This work
MoS_2	~145<br>(50 mA g^{-1})	~110<br>(50 mA g^{-1})	~90<br>(50 mA g^{-1})	85<br>(50 mA g^{-1})	Ref. S8
MoS_2 nanosheets	~190<br>(20 mA g^{-1})	~165<br>(20 mA g^{-1})	161<br>(20 mA g^{-1})	~160<br>(20 mA g^{-1})	Ref. S9
MoS_2/Graphene	~250<br>(25 mA g^{-1})	240<br>(25 mA g^{-1})	N/A	N/A	Ref. S10
N-doped porous carbon fibres	~350<br>(50 mA g^{-1})	~260<br>(50 mA g^{-1})	~250<br>(50 mA g^{-1})	243<br>(50 mA g^{-1})	Ref. S11
$Na_2Ti_3O_7$/Carbon black	~210<br>(17.8 mA g^{-1})	~165<br>(17.8 mA g^{-1})	~120<br>(17.8 mA g^{-1})	N/A	Ref. S12

Figure S7. Comparison of our prepared 3D MoS_2/rGO (MoS-G) with previously published SIB anode materials in terms of specific capacity and rate capability based on intercalation only.

References:

S1. Su, D., Dou, S. & Wang, G. Ultrathin MoS_2Nanosheets Anode Materials for Sodium-Ion Batteries with Superior Performance.*Adv. Energy Mater*. **5**, 1401205 (2014).

S2. Choi, S. H., Ko, Y. N., Lee, J. K. & Kang, C. Y. 3D MoS_2-Graphene Microspheres Consisting of Multiple Nanospheres with Superior Sodium Ion Storage Properties. *Adv. Funct. Mater*. **25**, 1780-1788 (2015).

S3. Xie, X., Ao, Z., Su, D., Zhang, J. & Wang, G. MoS_2/Graphene Composites Anodes with Enhanced Performance for Sodium-Ion Batteries: The Role the Two-Dimensional Hetero interface. *Adv. Funct. Mater*. **25**, 1393-1403 (2015).

S4. Wang, J., Luo, C., Gao, T., Langrock, A., Mignerey, A. C. & Wang, C. An advanced MoS_2/carbon anode for high-performance sodium-ion batteries. *Small* **11**, 473-481 (2015).

S5. Zhang, S., Yu, X., Yu. H., Chen, Y., Gao, P., Li, C. & Zhu, C. Growth of Ultrathin MoS_2Nanosheets with Expanded Spacing of (002) Plane on Carbon Nanotubes for High-Performance Sodium-Ion Battery Anodes. *Acs Appl. Mater. Interfaces* **6**, 21880-21885 (2014).

S6. M. Xu, F. Yi, Y. Niu, J. Xie, J. Hou, C. Cheng, S. Liu, W. H. Hu, Y. Li, C. M. Li, *J. Mater. Chem. A*, **2015**, DOI: 10.1039/C5TA00315F.

S7. Qin, W., Chen, T., Pan, L., Niu, L., Hu, B., Li, D., Li, J. & Sun, Z. MoS_2-reduced graphene oxide composites via microwave assisted synthesis for sodium ion battery anode with improve capacity and cycling performance. *Electrochim.Acta* **153**, 55-61 (2015).

S8. Park. J. *et al.* Discharge mechanism of MoS_2 for sodiumion battery: Electrochemical measurements and characterization. *Electrochim.Acta* **92**, 427-432 (2013).

S9. Bang, G. S., Nam, K. W., Kim, J. Y., Shin, J., Choi J. W. & Choi, S. Y. Effective Liquid-Phase Exfoliation and Sodium Ion Battery Application of MoS_2Nanosheets. *Acs Appl. Mater. Interfaces* **6**, 7084-7089 (2014).

S10. David, L., Bhandavat, R. & Singh, G. MoS_2/Graphene Composite paper for Sodium-Ion Battery Electrodes.*Asc Nano* **8**, 1759-1770 (2014).

S11. Fu, L., tang, K., Song, K., Aken, P. A. V., Yu, Y. & Maier, J. Natogen doped porous carbon fibres as anode material for sodium ion batteries with excellent rate performance. *Nanoscale* **6**, 1384- 1389 (2014).

S12. Rudola, A., Saravanan, K., Mason, C. W. & Balaya, P. $Na_2Ti_3O_7$: an intercalation based anode for sodium ion battery applications. . *J. Mater. Chem.A* **1**, 2653-2662 (2013).
